# Piceatannol Protects Human Retinal Pigment Epithelial Cells against Hydrogen Peroxide Induced Oxidative Stress and Apoptosis through Modulating PI3K/Akt Signaling Pathway

**DOI:** 10.3390/nu11071515

**Published:** 2019-07-04

**Authors:** Yiming Hao, Jie Liu, Ziyuan Wang, Liangli (Lucy) Yu, Jing Wang

**Affiliations:** 1 China-Canada Joint Lab of Food Nutrition and Health (Beijing), Beijing Technology & Business University (BTBU), Beijing 100048, China.; 2 Beijing Advanced Innovation Center for Food Nutrition and Human Health, Beijing Technology & Business University, Beijing 100048, China; 3 Department of Nutrition and Food Science, University of Maryland, College Park, MD 20742, USA

**Keywords:** piceatannol, ARPE-19 cell, oxidative stress, apoptosis, PI3K/Akt signaling pathway

## Abstract

This study investigated the protective effect and the molecular mechanism of piceatannol on hydrogen peroxide (H_2_O_2_)-induced retinal pigment epithelium cell (ARPE-19) damage. Piceatannol treatment significantly inhibited H_2_O_2_-induced RPE cell death and reactive oxygen species (ROS) generation by 64.4% and 75.0%, respectively. Results of flow cytometry showed that H_2_O_2_-induced ARPE-19 cells apoptosis was ameliorated by piceatannol supplementation, along with decreased relative protein expressions of Bax/Bcl-2, Cleave-Caspase-3, and Cleave-PARP. Moreover, piceatannol treatment induced NF-E2-related factor 2 (Nrf2) signaling activation, which was evidenced by increased transcription of anti-oxidant genes, glutamate-cysteine ligase catalytic subunit (GCLc), SOD, and HO-1. Knockdown of Nrf2 through targeted siRNA alleviated piceatannol-mediated HO-1 transcription, and significantly abolished piceatannol-mediated cytoprotection. LY294002 (PI3K inhibitor) dramatically blocked piceatannol-mediated increasing of Nrf2 nuclear translocation, HO-1 expression, and cytoprotective activity, indicating the involvement of PI3K/Akt pathway in the cytoprotective effect of piceatannol. The results from this suggest the potential of piceatannol in reducing the risk of age-related macular degeneration.

## 1. Introduction

Age-related macular degeneration (AMD) can cause irreversible vision loss which is a major threat for eye health in aging populations [1,2,3]. AMD is characterized by the degeneration of the macular retinal pigment epithelial (RPE) cells. Excessive reactive oxygen species (ROS) is one of the major risk factors for AMD, which causes oxidation of key cellular components and severe damage to local retinal pigment epithelium (RPE) cells pathophysiologically. Clinical studies revealed that antioxidants and zinc-containing supplements could dramatically reduce AMD progression rate [4]. Kim et al. reported that increased consumption of fruits and vegetables containing antioxidant nutrients and phytochemicals might provide some protection against AMD [5]. Growing evidence shows that early AMD protection and treatment should be focused on rescuing retinal pigment epithelial (RPE) cells from oxidative damage [4].

Nuclear factor erythroid 2-like 2 (Nrf2) is one of the most essential transcription factors in modulating many antioxidant genes. When the cells were exposed to an oxidative stress environment, Nrf2 could transfer to the nucleus and bind to the antioxidant response element (ARE), which could increase the expression of the downstream phase II antioxidant enzymes [6]. Nrf2 was modulated by PI3K/Akt signaling pathway which also played a crucial role in cell proliferation and apoptosis. Akt-Nrf2 activation was thought to be an efficient factor involved in AMD and other metabolism diseases which had been evidenced to modulate oxidant-induced RPE cell death [7].

Piceatannol (3,3′,4,5′-trans-tetrahydroxystilbene) is a natural stilbene found in fruits [8], seeds [9], and wine [10]. Piceatannol has been reported to have several beneficial properties, such as anti-oxidation [11], anti-inflammation [12], and neuroprotective activity [13]. The number and substituted position of hydroxys make piceatannol more preferentially active than other natural stilbene, such as resveratrol and oxyresveratrol [11]. Our previous study showed that piceatannol supplementation could inhibit TNF-α mediated inflammation and insulin resistance in 3T3-L1 adipocytes by modulating NF-κB and JNK/MAPK signaling pathways [14]. Lu et al. reported that piceatannol could protect ARPE-19 cells against vitamin A dimer-mediated photo-oxidative damage through activation of Nrf2/NQO1 signaling [15]. However, the mechanism underlying its protective effects has not been fully understood, especially the possible roles of PI3K/Akt signaling pathways in protecting RPE cells against oxidative stress and apoptosis.

In this study, the protective effect of piceatannol on hydrogen peroxide induced RPE cells’ oxidative damage and apoptosis, and the possible underlying mechanism(s) were investigated. The results from this work may shed new light on the potential role of piceatannol in reducing the risk of age-related macular degeneration.

## 2. Materials and Methods

### 2.1. Reagents, Chemicals and Antibodies

**Figure 1:**
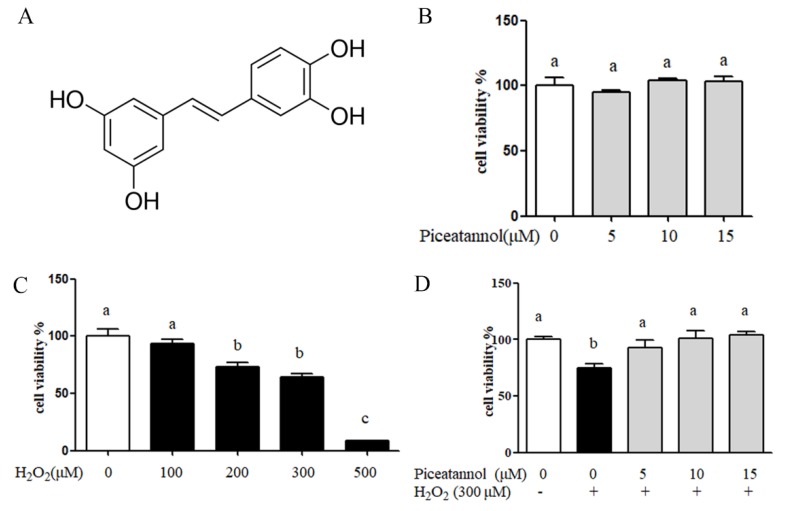
The cytoprotection of piceatannol induced by H_2_O_2_ in retinal pigment epithelium (ARPE-19) cells. ARPE-19 cells were seeded in a 96-well plate for 24 h, then treated with piceatannol for 24 h and induced with or without H_2_O_2_ for another 24 h. (**A**) The chemical structure of piceatannol. (**B**) The cytotoxicity of piceatannol in ARPE-19 cells. (**C**) The cell viability of ARPE-19 cells induced by different concentration of hydrogen peroxide. (**D**) The cytoprotection of piceatannol in ARPE-19 cells. + indicates with H_2_O_2_, - indicates without H_2_O_2_. Different letters present *p* < 0.05 calculated by ANOVA for each group.

LY294002 (ab146593) and the antibodies against α-tubulin (ab52866), Nrf2 (ab137550), HO-1 (ab13248), Akt (ab179463), p-Akt (ser 473) (ab81283), Catalase (ab16731), and GCLc (ab80841) were purchased from Abcam (Cambridge, UK). Histone H3 (#4499), Caspase 3 (#9662), cleave PARP (#5625), Bax (#2772), and Bcl-2 (#2872) were purchased from CST (MA, USA). siRNA (control siRNA sc-37007, Nrf2 siRNA sc-37030) were purchased from Santa Cruz Biotech (CA, USA). The piceatannol was purchased from Sigma (purity >95%) and dissolved in DMSO before use (Sigma Aldrich, St. Louis, MO, USA), and its chemical structure is presented in Figure 1A.

### 2.2. Cell Culture

ARPE-19 cells (human retinal pigment epithelial cell line, ATCC) were cultured in Dulbecco’s Modified Eagle Medium: Nutrient Mixture F-12 (DMEM/F-12) supplemented with 10% fetal bovine serum, 100 U/mL penicillin, and 100 μg/mL streptomycin (Gibco, Madison, USA). The cells were incubated at 37 °C with 5% CO_2_.

### 2.3. Cell Viability Assay

ARPE-19 cells (1 × 10^4^ cells/well) were plated in 96-well plates for 24 h, then treated with piceatannol and H_2_O_2_ separately for 24 h. After the treatment, the cell medium was replaced by equal volume of DMEM/F12 serum-free medium containing 0.5 mg/mL 3-(4,5-dimethyl-2-thiazolyl)-2,5-diphenyl-2-H-tetrazolium bromide (MTT, Sigma Aldrich, St. Louis, MO, USA) and incubated with ARPE-19 cells at 37 °C for 4 h. The supernatant was removed and the crystal violet was dissolved in DMSO. The absorbance at 490 nm was measured with a microplate reader (SPRAK 10M, Tecan, Männedorf, Switzerland).

### 2.4. Intracellular Reactive Oxygen Species (ROS) Measurement

The ARPE-19 cells (1 × 10^4^ cells/well) were plated in 96-well black plates for 24 h, and incubated with different concentrations of piceatannol for 24 h. Before H_2_O_2_ treatment, ARPE-19 cells were washed twice with Hank’s Balanced Salt Solution (HBSS) and incubated with 10 μM of DCFH-DA (Sigma Aldrich, St. Louis, MO, USA) in HBSS for 60 min at 37 °C. After HBSS washing, the ARPE-19 cells were induced by H_2_O_2_ in a serum free medium for 24 h. The fluorescence intensity was measured at an excitation wavelength of 475 nm and an emission wavelength of 530 nm with a microplate reader (SPRAK 10M, Tecan, Männedorf, Switzerland).

### 2.5. Western Blotting Assay

After treatment, the cells were washed three times with phosphate buffer saline (PBS). Then the ARPE-19 cells were lysised in Radio-Immunoprecipitation Assay (RIPA) lysis buffer supplemented with phosphatase inhibitor and protease inhibitor cocktails (Sigma aldrich, St. Louis, MO, USA) for 30 min on ice. After being centrifuged at 13,400× *g* for 20 min at 4 °C, the supernatant was obtained as total protein extracts. A nuclear and cytoplasmic protein extraction kit (Beyotime, Shanghai, China) was used to isolate the ARPE-19 cells’ nuclear protein by following the manufacturer’s instructions. Protein concentrations were determined with BCA protein assay kit (Thermo fisher, Madison, WI, USA). Protein samples were fractionated with 12% SDS-PAGE gel and then transferred to a PVDF membrane (Bio-Rad Laboratories, Hercules, USA). After blocking with 5% (*w*/*v*) skim milk in tris-buffered saline containing 0.1% Tween-20 (0.1% TBST) for 1.5 h at room temperature, the membrane was incubated with primary antibodies overnight at 4 °C, and secondary antibodies for 2 h at room temperature. Protein bands were visualized by using a chemiluminescence reagent and analyzed with a Bio-Rad imaging system (Bio-Rad Laboratories, Hercules, USA).

### 2.6. siRNA Interference

ARPE-19 cells (1 × 10^5^ cells/well) were seeded in 12-well plates for 24 h and then transfected with control siRNA or Nrf2 siRNA (100 μM) carried by lipofectamine 2000 (Thermo Fisher, Madison, USA) for 12 h. After transfection, ARPE-19 cells were incubated with piceatannol for 12 h before H_2_O_2_ treatment. The cytoprotection of piceatannol was evaluated by Western blotting or MTT assay.

### 2.7. Flow Cytometry

For the cell apoptosis assay, an Annexin V-fluorescein isothiocyanate (FITC)/propidium iodide (PI) apoptosis detection kit (Sigma Aldrich, St. Louis, MO, USA) was used according to the manufacturer’s protocols. ARPE-19 cells (2 × 10^5^ cells/well) were seeded in 6-well plates for 24 h and then incubated with or without 15 μM piceatannol for 24 h. After H_2_O_2_ treatment, the ARPE-19 cells were harvested and centrifuged at 1000 rpm for 5 min. After PBS washing three times, the cells (1 × 10^6^ cells/mL) were re-suspended by 1× binding buffer. Then, the cells were mixed gently with 5 μL of Annexin V-FITC and incubated for 30 min at 37 °C. After that, 10 μL of Propidium Iodide (PI) staining solution was added to the cells at room temperature for 10 min in a dark place. The Annexin V-FITC-positive and PI-negative cells were recorded as apoptotic cells, and the percentage of apoptotic cells in each group was recorded and analyzed with a CytoFLEX flow cytometer (Beckman Coulter, Inc.).

### 2.8. Coimmunoprecipitation (co-IP) Assay

ARPE-19 cells were incubated with piceatannol for 24 h and followed by treatment with H_2_O_2_ for 0.5 h. Then, the cells were lysed as described previously, and coimmunoprecipitation was operated according to the manufacturer’s protocol. Briefly, extracted proteins were immunoprecipitated with 5 μL Keap1 antibody for 12 h at 4 °C, and 20 μL protein A+G agarose beads (Beyotime, Shanghai, China) were added and incubated for another 4 h at 4 °C. After the supernatant was removed, the collected agarose beads were washed with 100 μL PBS (0.01 M, pH 7.4) 5 times and denatured by boiling for 5 min. The levels of relative protein expression were determined by Western blotting.

### 2.9. Statistical Analysis

Results were presented as the means ± standard deviation (SD). All statistical analyses were performed using SPSS software (version 20; IBM, Corp., Armonk, NY, USA). The differences among groups were assessed by Student’s t test or one-way analysis of variance followed by a Duncan’s post hoc test. *P* values less than 0.05 are considered to be statistically significant.

## 3. Results

### 3.1. Piceatannol Inhibited H_2_O_2_-Induced ARPE-19 Cells Death

The effect of hydrogen peroxide and piceatannol on ARPE-19 cells’ viability was evaluated by MTT assay. The results showed that piceatannol treatment had no significant adverse effect on RPE cells’ viability at a dose level of 5–15 μM (Figure 1B). H_2_O_2_ treatment dose dependently decreased ARPE-19 cells’ viability, and 300 μM H_2_O_2_ was used in the subsequent experiments (Figure 1C). As shown in Figure 1D, 300 μM H_2_O_2_ treatment significantly reduced 25.6% of RPE cells’ viability compared with the control group. However, the addition of piceatannol showed significant cytoprotection against H_2_O_2_ induced oxidative damage, and improved the cells’ viability by 18.5%, 26.6% and 29.5% at the concentrations of 5, 10 and 15 μM, respectively.

### 3.2. Piceatannol Repressed H_2_O_2_-Induced ARPE-19 Cells Apoptosis

**Figure 2:**
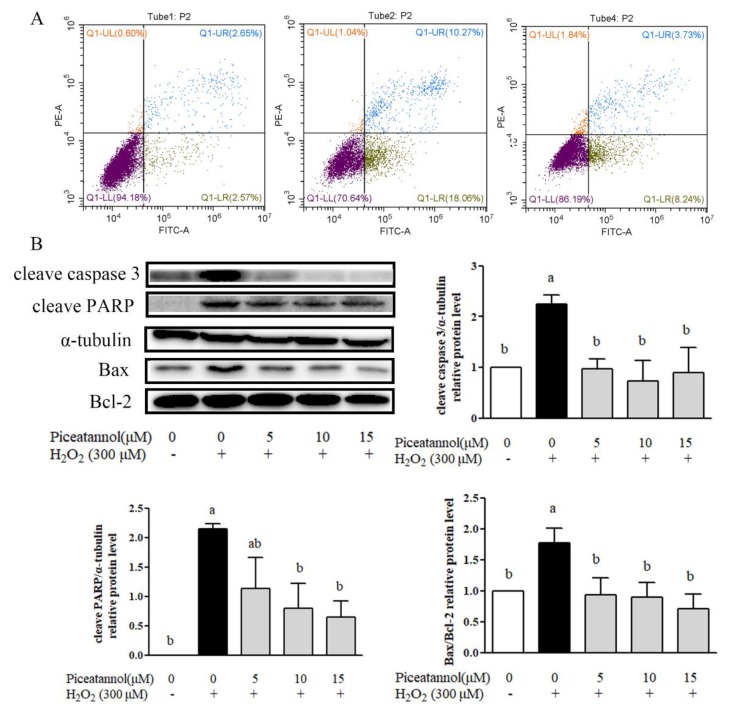
Piceatannol repressed H_2_O_2_-induced ARPE-19 cells apoptosis. The ARPE-19 cells were seeded in 6-well plates for 24 h, then treated with piceatannol for 24 h, and induced by H_2_O_2_ for 4 h. (**A**) Apoptosis was analyzed by being double stained with PI and Annexin V. (**B**) Apoptosis related protein (Cleave Caspase 3, Cleave PARP, Bax, Bcl-2) was detected by Western blotting. + indicates with H_2_O_2_, - indicates without H_2_O_2_. Different letters present *p* < 0.05 calculated by ANOVA for each group.

The protective effect of piceatannol against H_2_O_2_ induced ARPE-19 cells’ apoptosis was investigated by Annexin V-FITC and PI staining through flow cytometry. As shown in Figure 2A, 300 μM H_2_O_2_ treatment increased the apoptosis in ARPE-19 cells compared with the control group by 18%, whereas the treatment with piceatannol attenuated the cells’ apoptosis by about 10%. Moreover, piceatannol inhibited apoptosis related protein expressions induced by hydrogen peroxide. Results of Western blotting showed that piceatannol treatment significantly repressed the protein expression level of cleave caspase-3, cleave PARP and Bax/Bcl-2 (Figure 2B).

### 3.3. Piceatannol Exhibited Potent Anti-Oxidative Activities in ARPE-19 Cells Treated with H_2_O_2_

**Figure 3:**
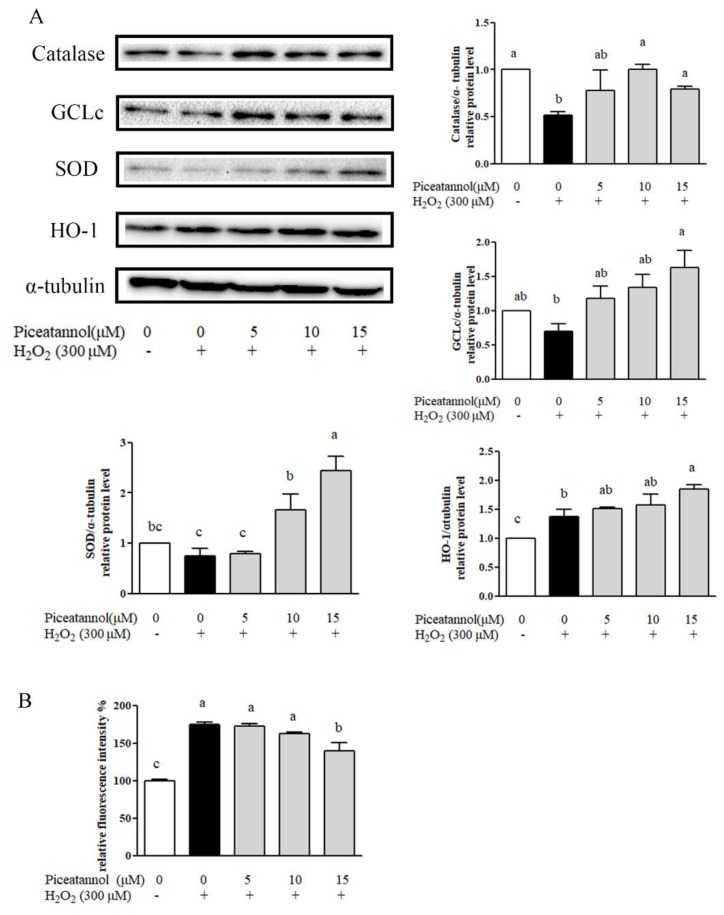
Piceatannol increased the expression of antioxidase in ARPE-19 cells. (**A**) The level of relative protein expression of Catalase, GCLc, SOD, HO-1 was determined by Western blotting. The ARPE-19 cells were incubated with piceatannol for 24 h and induced by H_2_O_2_ for 4 h. (**B**) The reactive oxygen species (ROS) production of piceatannol against H_2_O_2_ on ARPE-19 cells. + indicates with H_2_O_2_, - indicates without H_2_O_2_. Different letters present *p* < 0.05 calculated by ANOVA for each group.

The anti-oxidative capacity of piceatannol against oxidative stress in ARPE-19 cells was examined as was its effect on the expression of Phase II metabolizing enzymes including catalase, GCLc, SOD, and HO-1. Piceatannol (15 μM) was able to significantly enhance the expression of catalase, GCLc, SOD, and HO-1 (Figure 3A). Moreover, the content of reactive oxygen species (ROS), which reflected the cells’ oxidative status was also determined. As shown in Figure 3B, H_2_O_2_ treatment significantly elevated intracellular ROS production compared with the control group. On the contrary, the ROS elevation induced with H_2_O_2_ was effectively reversed by piceatannol treatment (Figure 3B). These results suggested that the cytoprotective effects of piceatannol were partly dependent on its anti-oxidative capacity.

### 3.4. Piceatannol Reduced H_2_O_2_-Induced RPE Cells’ Oxidative Damage through Nrf-2/HO-1 Signaling Pathway

**Figure 4:**
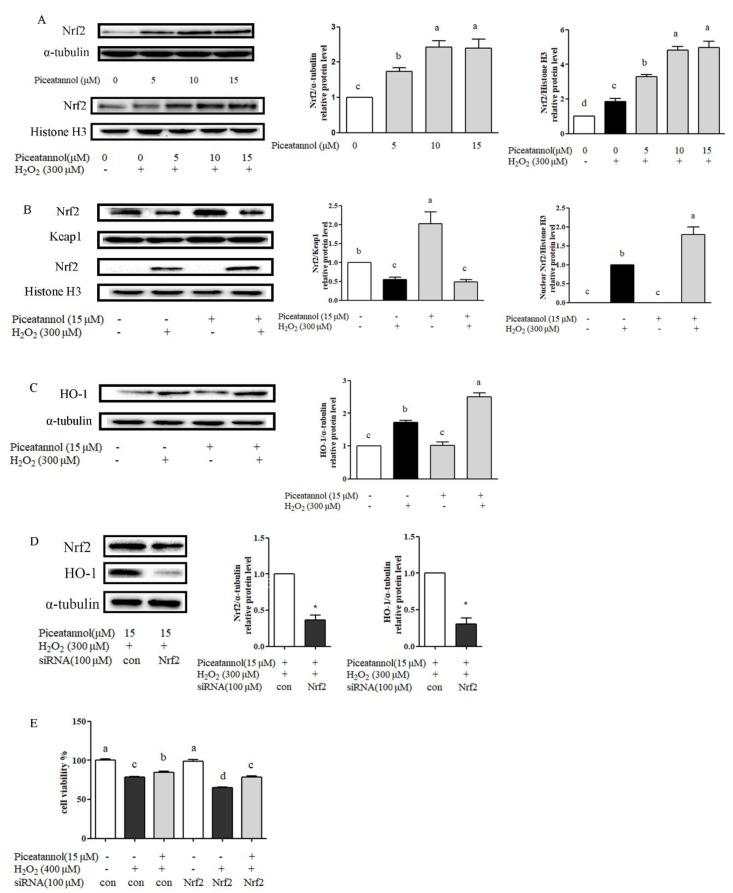
Piceatannol protected ARPE-19 cell damage induced by H_2_O_2_ via Nrf2/HO-1 signaling ­pathway. ARPE-19 cells were incubated with piceatannol for 24 h, then treated with or without 300 μM H_2_O_2_ for 0.5 h. (**A**) The level of relative protein expression of Nrf2 (total and nucleus) was determined by Western blotting. (**B**) The level of relative protein expression of Nrf2 and Keap1 was determined by Western blotting. (**C**) The level of relative protein expression of HO-1 was determined by Western blotting. ARPE-19 cells were treated with piceatannol for 24 h, then induced by 300 μM H_2_O_2_ for 4 h. (**D**) Protein expression level of Nrf2 and HO-1 was analyzed by Western blotting. ARPE-19 cells were transfected by siRNA for 12 h and then incubated with piceatannol for 12 h and induced by H_2_O_2_ for 4 h. (**E**) The cytoprotective effect of piceatannol was analyzed by MTT method. ARPE-19 cells were transfected by siRNA for 12 h and then incubated with or without piceatannol for 12 h and induced by H_2_O_2_ for 12 h. + indicates with H_2_O_2_ or piceatannol, - indicates without H_2_O_2_ or piceatannol. Different letters or * present *p* < 0.05 calculated by ANOVA or student’s *t* test for each group.

Western blotting results showed that the piceatannol-alone treatment could increase the total Nrf2 protein expression (Figure 4A). H_2_O_2_ treatment could improve Nrf2 protein nuclear transcription expression, which was also significantly increased after piceatannol treatment, compared with H_2_O_2_ control group in a dose related manner (Figure 4A). After H_2_O_2_ stimulation, thiol groups of Keap1 were oxidized or modified, resulting in the release and translocation of Nrf2 into the nucleus (Figure 4B). The protein expression of its downstream gene HO-1 was significantly increased compared with the blank group (Figure 4C). However, coimmunoprecipitation (Co-IP) assay showed that piceatannol treatment alone did not disrupt Nrf2-Keap1 association in cytosol (Figure 4B). In the absence of H_2_O_2_, piceatannol pre-treatment could not improve HO-1 expression. On the contrary, piceatannol could increase HO-1 expression obviously under oxidative stress (Figure 4C). Taking together, piceatannol addition could not increase Nrf2 protein nuclear translocation unless under the oxidative stress condition induced by H_2_O_2_.

To further testify these results, experiment on Nrf2 gene knockdown through targeted siRNA carried by lipofectamine 2000 was conducted. Results showed that the expression level of Nrf2 dramatically decreased in RPE cells after Nrf2 siRNA interruption (Figure 4D). Consequently, piceatannol-mediated high expression of HO-1 was almost abolished after Nrf2 siRNA knockdown (Figure 4D). In addition, Nrf2-siRNA interruption caused H_2_O_2_-induced cell death exacerbation, and counteracted the cytoptotective effect of piceatannol (Figure 4E). These results suggested that piceatannol could alleviate H_2_O_2_-induced RPE cells’ oxidant damage through Nrf2/HO-1 signaling pathway.

### 3.5. PI3K/Akt Signaling Pathway Played Vital Roles in Piceatannol against H_2_O_2_-Induced RPE Cells Oxidant Damage

**Figure 5:**
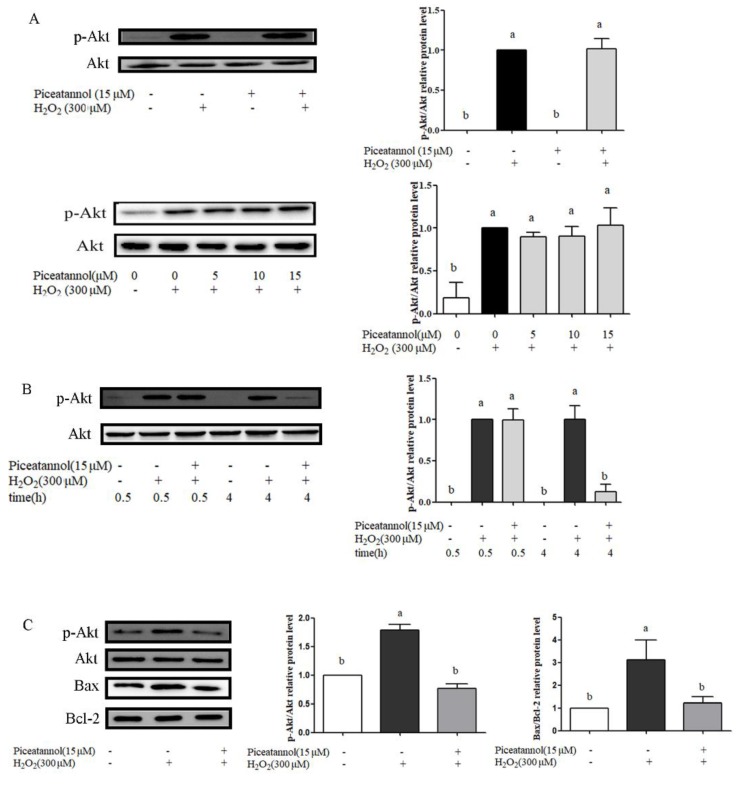
PI3K/Akt signaling pathway was activated in ARPE-19 cells induced by H_2_O_2_. (**A**) The protein expression of p-Akt (ser 473) and total Akt proteins in ARPE-19 cells was analyzed by Western blotting. APRE-19 cells were pre-treated with or without piceatannol for 24 h, followed by H_2_O_2_ treatment for 0.5 h. (**B**) The protein expression of p-Akt (ser 473) and total Akt proteins in ARPE-19 cells was analyzed by Western blotting. ARPE-19 cells were incubated with or without piceatannol for 24 h then induced by H_2_O_2_ for 0.5 and 4 h, respectively. (**C**) The protein expression of p-Akt (ser 473), total Akt, Bax and Bcl-2 were analyzed by Western blotting. ARPE-19 cells were incubated with or without piceatannol for 24 h then induced by H_2_O_2_ for 4 h. + indicates with H_2_O_2_ or piceatannol, - indicates without H_2_O_2_ or piceatannol. Different letters present *p* < 0.05 calculated by ANOVA for each group.

Some studies indicated that PI3K/Akt activation played a key role in cytoprotection under oxidative stress [16,17,18]. The effects of piceatannol on PI3K/Akt signaling pathway were investigated by Western blotting. Our results indicated that the piceatannol addition alone did not cause the phosphorylation of Akt protein expression. However, the result was reversed after exposure to H_2_O_2_ (Figure 5A). In the presence of H_2_O_2_, there is no significant difference between piceatannol treatment (5, 10 and 15 µmol/mL) and H_2_O_2_ positive control during the beginning 30 min (Figure 5A). However, piceatannol treatment significantly decreased phosphorylation of Akt protein expression compared with H_2_O_2_ control group at 4 h (Figure 5B). Further analysis showed that the expression of Bax/Bcl-2 treated by piceatannol was downregulated significantly compared with H_2_O_2_ treatment group, indicating that downregulation of p-Akt at 4 h was helpful for the anti-apoptosis ability of piceatannol (Figure 5C).

**Figure 6:**
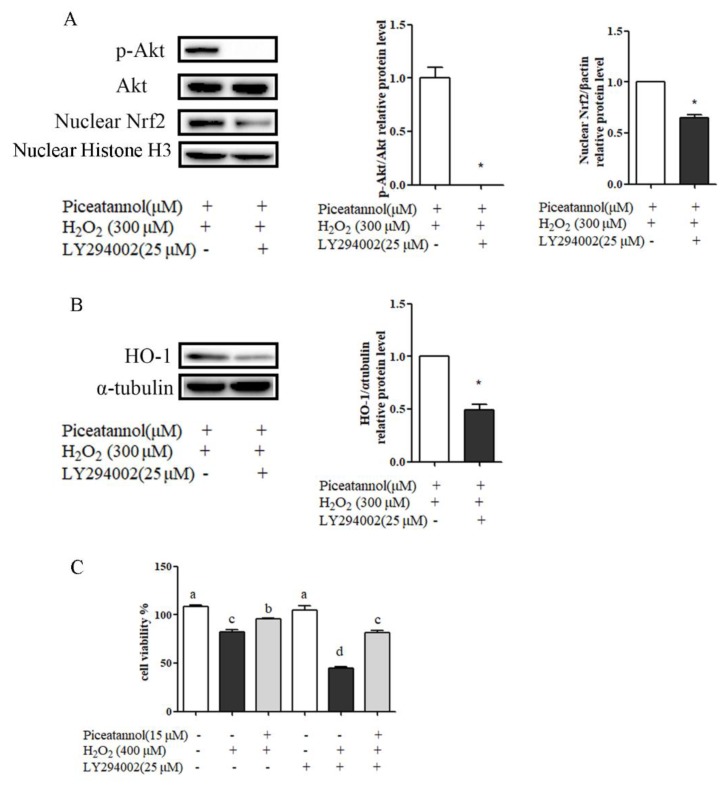
The PI3K/Akt signaling pathway was involved in RPE cells’ cytoprotection against oxidative stress and apoptosis. (**A**) ARPE-19 cells were incubated with piceatannol for 24 h, then incubated with LY294002 (25 μM) for 4 h, and induced by H_2_O_2_ for 0.5 h. The protein expression level of p-Akt (ser 473), total Akt proteins, and nucleus Nrf2 was analyzed by Western blotting. (**B**) The protein expression level of HO-1 was analyzed by Western blotting. ARPE-19 cells were incubated with piceatannol for 24 h, then pretreated with or without LY294002 (25 μM) for 4 h, and then induced by H_2_O_2_ for 4 h. (**C**) The cytoprotective effect of piceatannol was analyzed by MTT method. ARPE-19 cells were incubated with piceatannol for 24 h, then pretreated with or without LY294002 (25 μM) for 4 h, and induced by H_2_O_2_ for 24 h. + indicates with treatment, - indicates without treatment. Different letters or * present *p* < 0.05 calculated by ANOVA or student’s *t* test for each group.

To examine whether, and how, the PI3K/Akt signaling pathway is involved in cytoprotection against H_2_O_2_-induced RPE cells’ oxidant damage, PI3K/Akt inhibitor (LY294002) was used to illustrate the relationship between PI3K/Akt and Nrf2/HO-1 signaling pathways and cell apoptosis by Western blotting. As shown in Figure 6, LY294002 treatment blocked a piceatannol-mediated increase of Nrf2 nuclear translocation, HO-1 expression, and cytoprotective activity. These indicated that Akt activation was necessary for Nrf2 nuclear translocation and cytoprotective effect induced by piceatannol.

## 4. Discussion

In our present study, piceatannol was proven to induce the transcription of ARE-dependent genes (Catalase, GCLc, SOD and HO-1), while inhibiting H_2_O_2_-induced apoptosis and ROS accumulation in RPE cells. A previous study suggested that piceatannol could scavenge diverse free radicals, such as hydroxyl, superoxide, peroxyl, and lipid peroxyl radicals, and that the O-H and C-H groups play an important role in its anti-oxidative activities [19]. Thus, extensive antioxidant defense should be responsible for piceatannol cytoprotection. Moreover, accumulative ROS could induce RPE cells mitochondrial dysfunction and promote apoptosis in human RPE cells [20]. This study for the first time demonstrated that piceatannol supplementation attenuated proteins’ expression of Bax/Bcl-2, Cleave-Caspase-3, and Cleave-PARP. These results were in accordance with a previous report, which indicated that piceatannol protected ARPE-19 cells against vitamin A dimer-mediated photo-oxidative damage [15].

Furthermore, we demonstrated a novel mechanism contributing to piceatannol protection on RPE cells through the PI3K/Akt signaling pathway. Activation of PI3K/Akt can stimulate various biological responses, such as glucose transport [21], glycogen synthesis [22], and protein synthesis [23]. Our previous study showed that piceatannol could inhibit TNF-α-mediated inflammation and insulin resistance in 3T3-L1 adipocytes through Akt-dependent forkhead box O1 (FoxO1) signaling pathway [14]. Moreover, the PI3K/Akt signaling pathway has been evidenced to be involved in alleviating oxidative stress which could induce cell death and mitochondrial dysfunction [24,25,26]. A PI3K/Akt modulated anti-oxidant defense pathway such as Nrf2/HO-1 might play a critical role in RPE cells’ cytoprotection [25,27]. For example, Zhang et al. has reported that salvianolic acid A could protect RPE cells against oxidative stress through Akt phosphorylation [4], and Nrf2/HO-1 activation suggested that syringic acid protected retinal ganglion cells against H_2_O_2_-induced apoptosis through the activation of the PI3K/Akt signaling pathway [26]. Our present study also indicated that PI3K/Akt was involved in its cytoprotection against H_2_O_2_-induced cell damage (Figure 6C). PI3K/Akt inhibitor (LY294002) inhibited Nrf2 nuclear translocation and anti-oxidase expression, and abolished the cytoprotection of piceatannol (Figure 6A,B). These findings were consistent with the observation by Lu et al. that inhibition of Akt by LY294002 blocked piceatannol-mediated protection against photo-oxidation on ARPE-19 cells [15]. However, there are two new findings different from the previous study. Firstly, reports from Lu et al. suggested that piceatannol induced Nrf2 nuclear translocation, regardless of photo-oxidant treatment and total Nrf2 protein, was not significantly affected by piceatannol treatment [15]. Our result was the opposite of this conclusion, indicating that Nrf2 nuclear translocation was dependent on oxidative stress stimulation. As shown in Figure 4A,B, piceatannol treatment alone significantly increased total Nrf2 protein expression but did not induce their translocation to nuclei without the addition of H_2_O_2_. Secondly, previous studies also showed that protective actions of piceatannol could directly increase p-Akt expression regardless of oxidative stress stimuli. For example, piceatannol have been evidenced to cause neuroprotective activity as it could alleviate β-amyloid-induced cell apoptosis by activating p-Akt expression [13]. However, our present study indicated that piceatannol treatment alone could not increase p-Akt in ARPE-19 cells (Figure 5A), and the activation by H_2_O_2_ was necessary for subsequent Nrf2 translocation and antioxidative enzyme expression (Figure 6A,B). In addition, different from previous reports that piceatannol treatment could dose-dependently increase p-Akt compared with vitamin A dimer-mediated photo-oxidative group [15] our results showed that piceatannol treatment significantly decreased p-Akt compared with H_2_O_2_ group with 4 h treatment (Figure 5B). Thus, we suggest a novel hypothesis that the inhibition of p-Akt activation at 4 h could attenuate the apoptosis of ARPE-19 cells under oxidative stress, which was supported by the abolishment of relative apoptosis protein expression when treated with piceatannol (Figure 5C). The result was in agreement with Yan et al., and others, that the protein expression level of p-Akt was downregulated after treating with wogonin in the presence of H_2_O_2_ for 3 h, followed by decreased cell apoptosis [28].

## 5. Conclusions

Taking together, the PI3K/Akt signaling pathway played a vital role in piceatannol protection against H_2_O_2_-induced RPE cells oxidant damage. Firstly, sustained Akt activation induced by H_2_O_2_ is necessary for keap1/Nrf2 detaching, then more Nrf2 induced by piceatannol could translocate to nuclei and translate antioxidative enzymes to counteract oxidative stress. Secondly, decreased Akt activation caused by piceatannol could be helpful for reducing H_2_O_2_-induced RPE cell apoptosis.

This work demonstrated that piceatannol had a preventive function in H_2_O_2_-induced RPE cell damage and apoptosis through the PI3K/Akt signaling pathway. The results from this study may promote the development of piceatannol as a nutraceutical and potentially functional food ingredient for reducing the risk of age-related macular degeneration.
